# Bone Marrow Stromal Cell Transplantation Mitigates Radiation-Induced Gastrointestinal Syndrome in Mice

**DOI:** 10.1371/journal.pone.0024072

**Published:** 2011-09-15

**Authors:** Subhrajit Saha, Payel Bhanja, Rafi Kabarriti, Laibin Liu, Alan A. Alfieri, Chandan Guha

**Affiliations:** 1 Department of Radiation Oncology, Albert Einstein College of Medicine, Montefiore Medical Center, Bronx, New York, United States of America; 2 Department of Pathology, Albert Einstein College of Medicine, Montefiore Medical Center, Bronx, New York, United States of America; Ulm University, Germany

## Abstract

**Background:**

Nuclear accidents and terrorism presents a serious threat for mass casualty. While bone-marrow transplantation might mitigate hematopoietic syndrome, currently there are no approved medical countermeasures to alleviate radiation-induced gastrointestinal syndrome (RIGS), resulting from direct cytocidal effects on intestinal stem cells (ISC) and crypt stromal cells. We examined whether bone marrow-derived adherent stromal cell transplantation (BMSCT) could restitute irradiated intestinal stem cells niche and mitigate radiation-induced gastrointestinal syndrome.

**Methodology/Principal Findings:**

Autologous bone marrow was cultured in mesenchymal basal medium and adherent cells were harvested for transplantation to C57Bl6 mice, 24 and 72 hours after lethal whole body irradiation (10.4 Gy) or abdominal irradiation (16–20 Gy) in a single fraction. Mesenchymal, endothelial and myeloid population were characterized by flow cytometry. Intestinal crypt regeneration and absorptive function was assessed by histopathology and xylose absorption assay, respectively. In contrast to 100% mortality in irradiated controls, BMSCT mitigated RIGS and rescued mice from radiation lethality after 18 Gy of abdominal irradiation or 10.4 Gy whole body irradiation with 100% survival (p<0.0007 and p<0.0009 respectively) beyond 25 days. Transplantation of enriched myeloid and non-myeloid fractions failed to improve survival. BMASCT induced ISC regeneration, restitution of the ISC niche and xylose absorption. Serum levels of intestinal radioprotective factors, such as, R-Spondin1, KGF, PDGF and FGF2, and anti-inflammatory cytokines were elevated, while inflammatory cytokines were down regulated.

**Conclusion/Significance:**

Mitigation of lethal intestinal injury, following high doses of irradiation, can be achieved by intravenous transplantation of marrow-derived stromal cells, including mesenchymal, endothelial and macrophage cell population. BMASCT increases blood levels of intestinal growth factors and induces regeneration of the irradiated host ISC niche, thus providing a platform to discover potential radiation mitigators and protectors for acute radiation syndromes and chemo-radiation therapy of abdominal malignancies.

## Introduction

Accidental or intended radiation exposure in a mass casualty setting presents a serious and on-going threat. At radiation doses of 3 to 8 Gy, morbidity and lethality is primarily caused from hematopoietic injury and victims can be rescued by bone marrow transplantation (BMT). However, with exposure to larger doses, victims suffer irreversible hematopoietic and gastrointestinal injury and usually perish despite supportive care and BMT. While BMT may have some benefit in mitigating hematopoietic syndrome, currently there are no approved medical countermeasures to alleviate radiation-induced gastrointestinal syndrome (RIGS).

RIGS results from a dose-dependent, direct cytocidal and growth inhibitory effects of irradiation on the villous enterocytes, crypt intestinal stem cells (ISC) [1], [2], [3], the stromal endothelial cells [4] and the intestinal subepithelial myofibroblasts (ISEMF) [5]. Subsequent loss of the mucosal barrier results in microbial infection, septic shock and systemic inflammatory response syndrome. The cells in the ISC niche, consisting of micovascular endothelial cells, mesenchyme-derived ISEMF [5] and pericryptal macrophages [6] provide critical growth factor/signals for ISC regeneration and intestinal homeostasis [7]. Of these, ISEMF continuously migrate upward from the crypt base to the villous tip along with ISC and transit amplifying enterocytes, establishing signaling crosstalk and regulating ISC self-renewal and differentiation [5], [8]. ISEMF interacts with pericryptal macrophages with subsequent release of PGE2 that could reduce radiation-induced apoptosis of enterocytes [9], [10]. Pericryptal macrophages form synapses with crypt stem cells and secretes growth factors to stimulate ISC proliferation [6] upon activation of Toll-like receptors sensing the entry of bacteria and other intestinal pathogens.

Since RIGS results from a combination of radiation-induced loss of crypt progenitors and stromal cells along with aberrant signaling in the ISC niche, we rationalized that the acute loss of stromal cells in the ISC niche would require rapid compensation of their functions. This could be best achieved with cell replacement therapies that restore the ISC niche after irradiation so that the stromal cells can secrete growth factors and provide necessary signals for survival, repair and regeneration of the irradiated intestine. Earlier reports demonstrated that donor bone marrow-derived cells could contribute to multiple lineages in the gastrointestinal tract and facilitate intestinal regeneration in patients with graft-versus-host disease and ulcer [11] and in animal models of colitis [12]. Because of ease in cell culture and its ability to differentiate into multiple tissue lineages, transplantation of bone marrow-derived mesenchymal stem cells (MSC) has been a very attractive option for a wide range of clinical applications [13], such as, severe treatment-resistant graft-versus-host diseases of the gut [14]. Besides trans-differentiating into ISEMF and stimulating ISC proliferation, MSC transplantation has also been shown to reprogram host macrophages to induce an anti-inflammatory response and thereby minimizing sepsis in a murine model of colitis [15]. Intravenous injection of MSC resulted in enhanced engraftment in irradiated organs, including, small intestine with subsequent increase in the regeneration of the intestinal epithelium and accelerated recovery of the villi post-radiation in mice models [16]. Genetic modification of donor MSCs with superoxide dismutase [17] or CXCR4 [18] transgene augments the engraftment and mitigation of intestinal radiation injury. However, till date, transplantation of whole bone marrow or MSC has not been successful in ameliorating RIGS and improve survival of mice that received >10 Gy of irradiation in a single fraction [16], [17], [18]. We reasoned that the failure of cell-based therapies in ameliorating RIGS after lethal doses of irradiation is because of absence of important cellular components of the ISC niche, including endothelial cells and macrophages, in the donor MSC population. Since bone marrow could provide a source of all the major cell types in the ISC niche, namely, ISEMF, endothelial cells and macrophages, we amplified the stromal cell population by culturing freshly isolated bone marrow cells in mesenchymal basal medium and collected the adherent stromal cells for transplantation into mice exposed to lethal doses of whole body or abdominal irradiation. In this report, we demonstrate that bone marrow-derived adherent stromal cell transplantation (BMASCT), 24 hours following exposure to lethal AIR of 16–20 Gy, stimulated ISC regeneration, restored the functional integrity of the villi, dampened inflammatory response and mitigated RIGS in C57Bl/6 mice.

## Results

### BMASCT mitigates RIGS and improves survival of mice after lethal doses of irradiation

**Figure 1:**
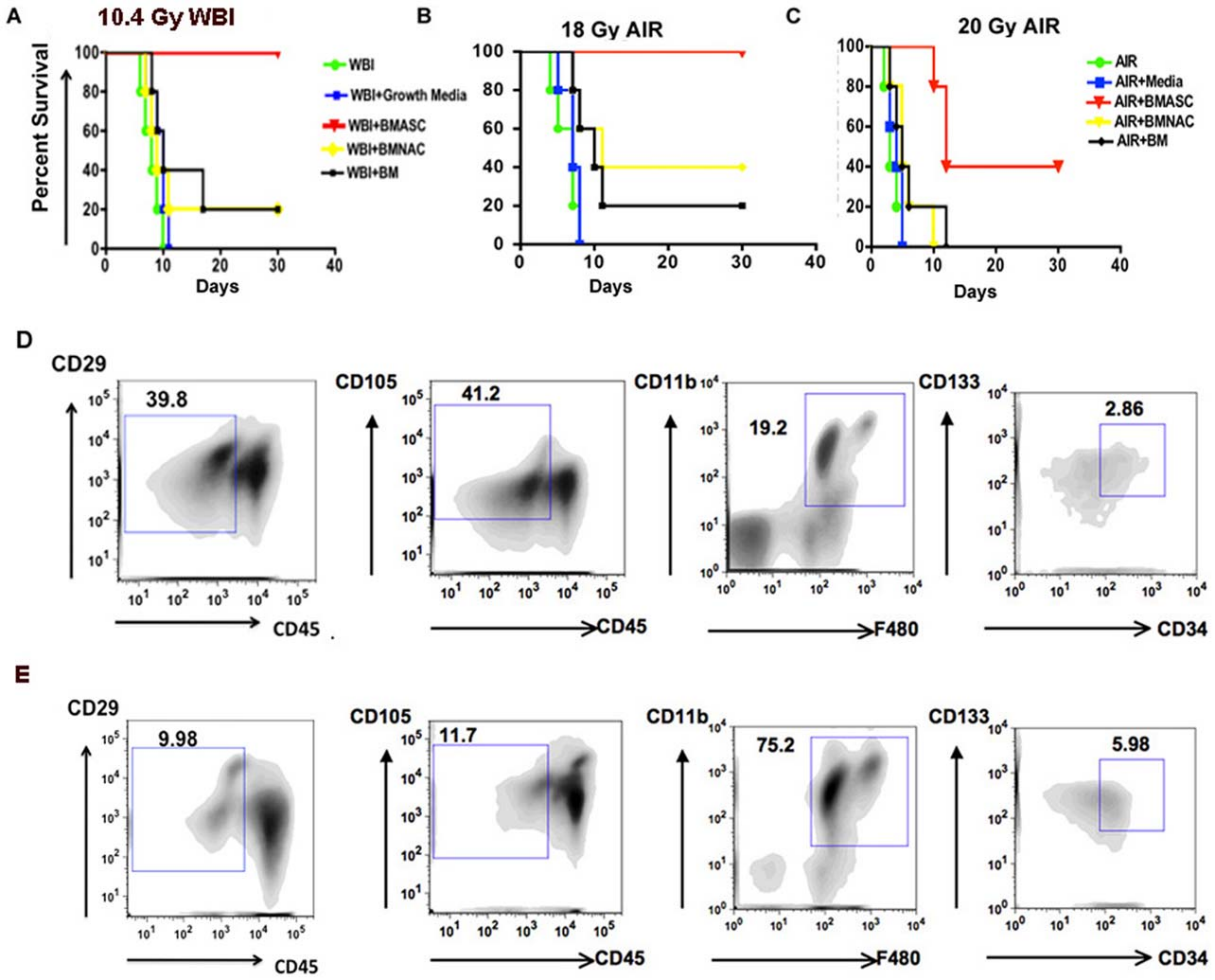
A–C. BMASCT improves survival of C57Bl/6 mice following AIR. Kaplan-Meier survival analysis of mice (n = 25) receiving BMASCT, 24 and 72 hrs after irradiation, showed 100% survival after (A) 10.4 Gy WBI (p<0.0006) and (B) 18 Gy AIR (p<0.0007); and 40% survival after (C) 20 Gy AIR (p<0.01). Whole bone marrow, BMNAC and culture media failed to improve survival. **D–E. Flow cytometric characterization.** (D) BMASC and (E) BMNAC population using MSC-specific (CD105+CD45−/CD29+CD45−), macrophage-specific (CD11b+F480+) and endothelial-specific (CD133+CD34+CD45−) markers.

Mortality from acute radiation syndromes results from dose-dependent radiation injury to various organs. While BMT is effective in improving survival of mice exposed to doses up to 8–9 Gy, it is relatively ineffective as the sole treatment with higher doses of exposure. We have previously demonstrated that a whole body exposure of ≥10.4 Gy induces RIGS and 100% mortality within 10–15 days in C57Bl/6 mice [1]. In order to confirm that RIGS is induced after exposure to a single fraction of Whole Body Irradiation (WBI) of 10.4 Gy, we examined whether BMT can improve the survival of C57Bl/6 mice. While 100% of the untreated animals died within 10 days, animals receiving BMT had only 20% survival (Fig. 1A), indicating that whole marrow that contained primarily CD45+ve hematopoietic cells (Figure S1) failed to rescue these animals from RIGS. We, then, examined whether transplantation of bone marrow-derived stromal cells that have been enriched for MSC, Endothelial Progenitor Cell (EPC) and macrophages upon culture in mesenchymal basal medium could mitigate radiation injury in these animals. Fig. 1A demonstrates that BMASCT rescued 100% of the irradiated animals (p<0.0009), indicating that stromal cell therapy may provide factors to repair and regenerate the intestinal epithelium damaged by irradiation.

To limit the exposure of the bone marrow to irradiation while escalating the dose to intestine, we delivered Abdominal Irradiation (AIR) (12–20 Gy) after shielding the thorax, head and neck and extremities, as described previously [19], [20]. AIR did not significantly impact the peripheral blood count at day 5 (Figure S2) post-exposure, indicating that the bone marrow was not severely damaged by AIR. Control animals that received either, PBS, or culture medium died within 10 days after exposure to AIR≥16 Gy with characteristic signs and symptoms of RIGS, including, diarrhea, black stools and weight loss. In contrast, animals that received AIR+BMASCT had well-formed stools, maintained body weight (24.1±0.7 g in AIR+BMASCT versus 16.21±1.8 g in AIR cohort, p<0.001) and had 100% survival beyond 25 days (18 Gy AIR, p<0.0007, Fig. 1B). At 20 Gy, BMASCT rescued 40% of the animals with survival greater than 25 days, while irradiated animals without BMASCT died within 5 days (median survival time of AIR cohort, 3±0.5 d versus AIR+BMASCT cohort, 12±1.8 d; p<0.01, Fig. 1C). Transplantation of CD45+ hematopoietic cell-enriched bone marrow derived non-adherent cell (BMNAC) and whole bone marrow cells failed to rescue AIR-treated mice (Fig. 1B–C,E & Figure S1), indicating that stromal cells were responsible for the salvage of RIGS.

### Both myeloid and non-myeloid cell populations are needed for RIGS mitigation

**Figure 2:**
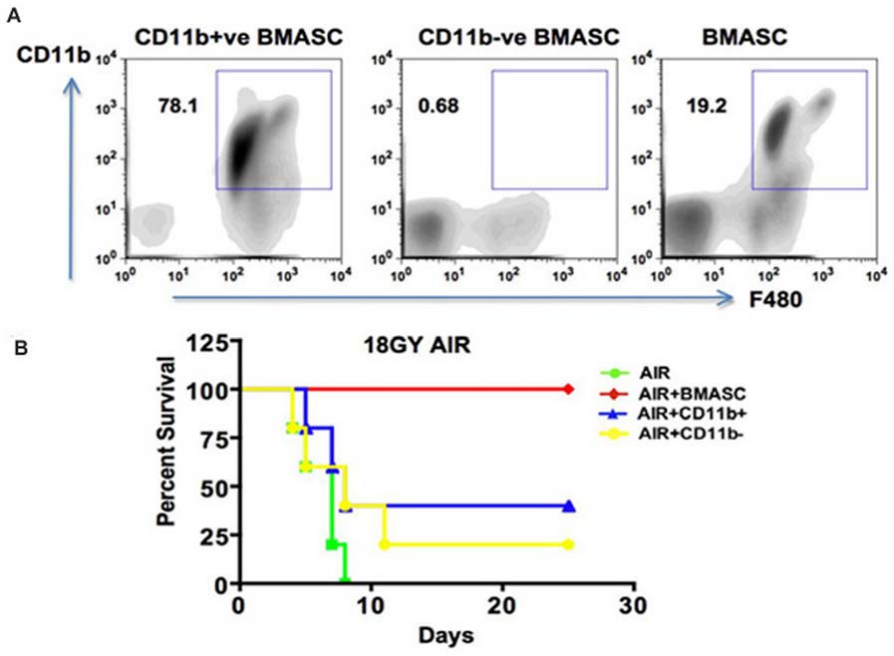
Both myeloid and non-myeloid fractions of BMASC are needed for RIGS mitigation. **A.** Flow cytometry of macrophage population in CD11b+ and CD11b− BMASC. **B.** Kaplan-Meier survival analysis.

Flow cytometric analysis of donor cells demonstrated that Bone marrow-derived adherent stromal cell (BMASC) population included, primarily MSCs (CD105+CD45− 41.2%±1.8; CD29+CD45− 39.8%±1.2), macrophages (CD11b+F480+ 19.2%±1.2) and EPCs (CD133+CD34+CD45−2.6%±0.89) and CD45+ hematopoietic cells (Fig. 1D). CD44 and Sca1 staining further confirmed the presence of MSC population (Figure S3). To evaluate the individual roles of CD11b+ macrophage-enriched cells versus CD11b− MSC-enriched stromal cell fraction (Figure S4) in RIGS mitigation, BMASC population was fractionated by cell sorting using anti-CD11b-magnetic beads, followed by transplantation 24 hrs post-AIR. Transplantation of either the macrophage-enriched (78.1%±2.8 F480+ cells), MSC-deficient (<1.5% CD105+ve cells) CD11b+ve BMASC or macrophage-deficient (0.68%±0.03 F480+ cells), MSC-enriched (68–71% CD105+ve) CD11b−ve BMASC cell population mitigated only 30–40% of the animals irradiated with 18 Gy AIR (Fig. 2A–B, Figure S4). Survival was salvaged to 100% when the CD11b+ and CD11b− populations were admixed and transplanted, indicating that the combination of macrophages and bone marrow stromal cells, including MSC and EPC fractions was necessary for RIGS mitigation.

### BMASCT induces structural and functional regeneration of intestine

**Figure 3:**
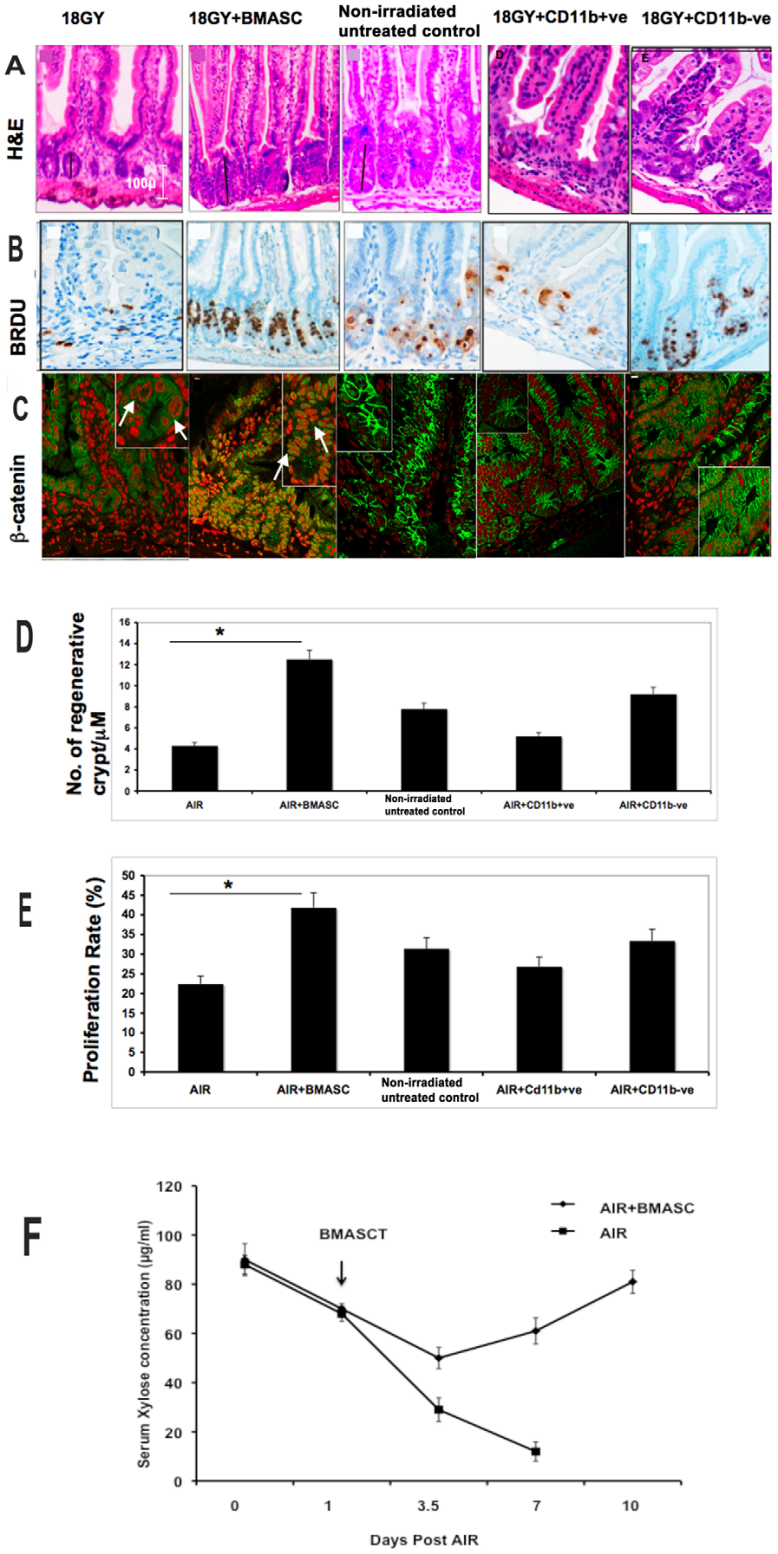
BMASCT mitigates RIGS by promoting structural and functional regeneration of the irradiated intestine. **A.** H&E staining, **B.** Brdu immunohistochemistry, **C.** β-catenin immunohistochemistry. β-catenin stained green and nucleus was stained with DAPI (pseudo colored with red). Confocal microscopic images (63×) were magnified 2.3× (inset). Note the greater crypt depth (A), increase in crypt cell proliferation (B) and an increase in nuclear translocation of β-catenin (stained yellow) in AIR+BMASCT cohort compared to other cohorts. **D.** Number of regenerative crypts, **E.** Crypt proliferation rate and **F.** Xylose absorption assay. A time course study showed significant recovery (p<0.0003) of xylose absorption at 7 days post-irradiation in AIR+BMASCT-treated animals compared to the AIR cohort.

Histomorphological evaluation after hematoxylin-eosin staining demonstrated that the AIR+BMASCT-treated animals exhibited an increase in the overall size of the crypts and maintained villous length (Fig. 3A, Figure S5 & S10). The percentage of the BrdU+ve crypt epithelial cells synthesizing DNA was significantly enhanced in this cohort of mice at 3.5 days post-irradiation (AIR+BMASCT, 42.82±2.01 versus AIR, 23.43±1.66; P<0.04; Fig. 3B and E). The numbers of regenerative crypt microcolonies per unit intestinal cross sectional area at 3.5 days post-irradiation serves as a surrogate indicator of crypt regenerative response post-irradiation [1], [21], [22]. The crypt microcolony count was increased significantly in AIR+BMASCT cohort, compared with those that received AIR alone (AIR+BMASCT, 12.5±1.2/µm versus AIR, 6.8±0.8, p<0.004, Fig. 3D), indicating intestinal regenerative response following BMASCT. Consistent with the regenerative response, immunohistological analysis demonstrated the presence of nuclear β-catenin in the AIR+BMASCT-treated animals, while cytosolic staining was predominant in the animals receiving AIR (Fig. 3C), suggesting that BMASCT activates the Wnt β-catenin pathway in crypt cells to stimulate proliferation post-irradiation.

We performed xylose absorption test and determined the functional recovery of the intestinal villi in RIGS. Since xylose is not metabolized in the body, serum xylose level is a good indicator of the intestinal absorptive capacity in animals fed with a test dose of xylose [1]. Compared to animals that received AIR alone, xylose absorption was significantly improved in animals that received BMASCT at 7 d post AIR (AIR+BMASCT, 72±5.5 g/ml vs. AIR, 35±2.7 g/ml; p<0.004; Fig. 3F), indicating quick functional restitution of the intestinal villi.

### BMASCT promotes survival of irradiated Lgr5-positive crypt base columnar cells

**Figure 4:**
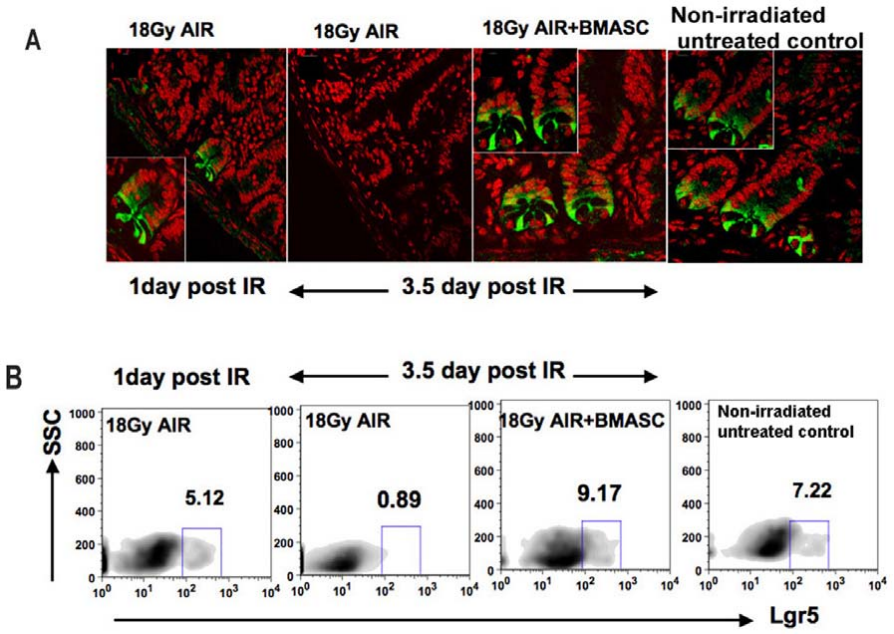
BMASCT promotes survival of *Lgr5*-positive crypt base columnar cells following AIR. **A.** Confocal microscopic imaging of EGFP expression in the jejunum of Lgr5-EGFP-ires-CreERT2 transgenic mice. *Lgr5*-EGFP+ve crypt cells are present at 1 d post-AIR but are absent at 3.5 d post-AIR, indicating the time course of radiation-induced ISC death. BMASCT inhibits the radiation-induced cell loss of *Lgr5+*ISCs. Confocal microscopic images (63×) were magnified 2.3× (inset). Nucleus was stained with DAPI and pseudo colored with red. **B.** Flow cytometric analysis of EGFP expression in crypt cells of Lgr5-EGFP-ires-CreERT2 transgenic mice post-AIR.

We examined the effect of AIR on the number of Lgr5-EGFP+ve crypt base columnar cells, the putative ISC population [3], [23], in the jejunum of *Lgr5-EGFP-IRES-creERT2* transgenic mice by detecting EGFP expression using confocal microscopy. While these cells are present at 1 d post-AIR, they are absent at 3.5 d post-AIR (Fig. 4A). Flow cytometric analysis confirmed the gradual loss of Lgr5+ve crypt ISCs following irradiation exposure (5.17%±1.8 at 1 d vs. 0.89%±0.15 at 3.5 d; p<0.001; Fig. 4B). In contrast, BMASCT increased the number of Lgr5-EGFP+ve CBCs at 3.5 d post-AIR (Fig. 4A). Flow cytometric analysis confirmed that BMASCT increased the number of irradiated Lgr5-GFP+ve crypt cells at 3.5 d post-AIR (9.27%±1.75, vs. 0.89%±0.15; (p<0.0003; Fig. 4B), possibly by providing signals for survival and growth. This provides us with a potential window of radiation mitigation, whereby BMASCT rescued lethally irradiated mice within 24 hrs of irradiation, but not after 72 hrs (Figure S6).

### BMASCT restores the ISEMF and pericryptal macrophages in the irradiated ISC niche

**Figure 5:**
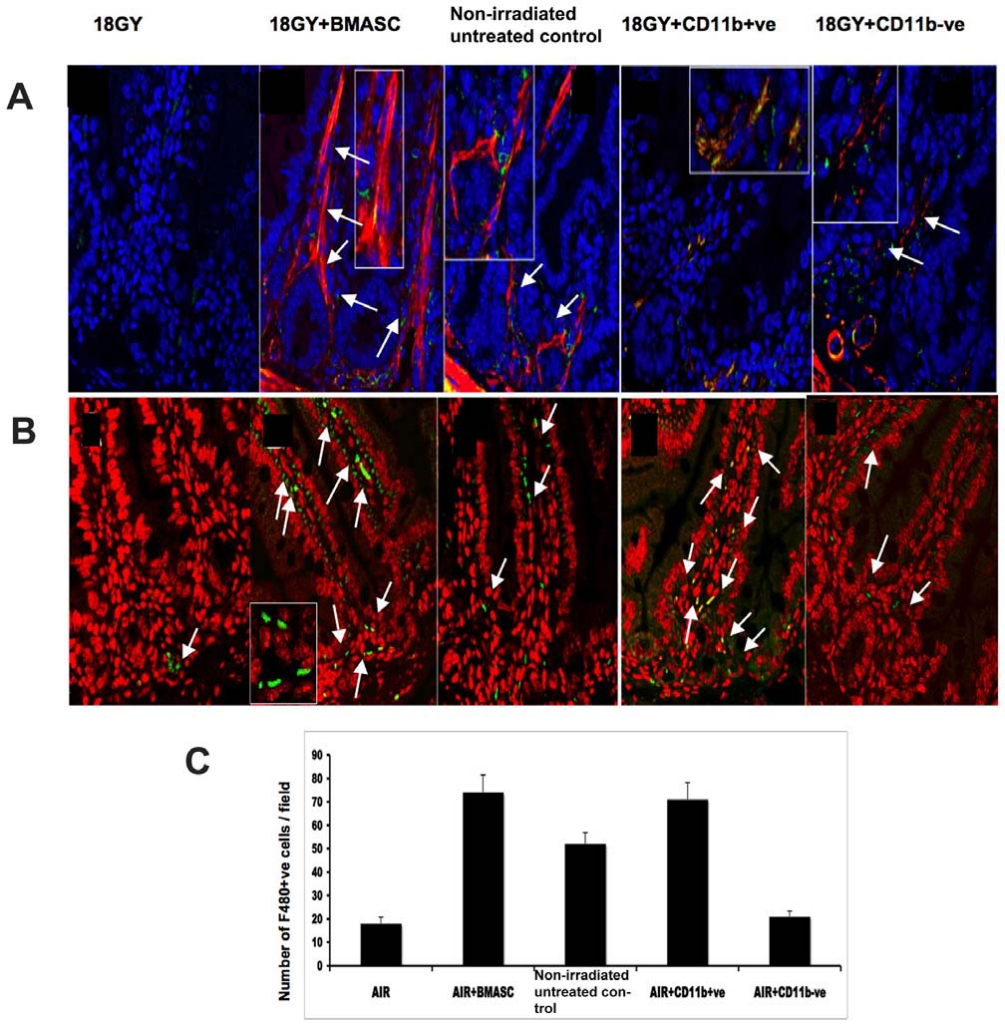
BMASCT restores the ISEMF and pericryptal macrophages of the ISC niche, 3.5 days post-AIR. **A.** ISEMF detection by immunohistochemistry and confocal microscopy using anti-α-SMA (stained red, indicated with arrow) and anti-desmin (stained green) antibodies. α-SMA+ve and desmin−ve ISEMF were reduced in AIR-treated animals, which was restored by BMASCT. Nucleus was stained with DAPI (blue). **B.** F480 Immunhistochemistry and confocal microscopic analysis and **C.** Quantification of Number of pericryptal macrophages. The number of F480+ve macrophages (green, indicated with arrow) increased at 3.5 d post-AIR in the AIR+BMASCT (p<0.003) and CD11b+ve BMASCT (p<0.006) group, compared to the AIR cohort, respectively. Nucleus was stained with DAPI (pseudo colored with red). Confocal microscopic images (63×) were magnified 2.3× (inset).

ISEMF and pericryptal macrophages provide the epithelial–mesenchymal cross-talk signals for growth, differentiation and cell fate determination to ISCs [6], [8], [9]. Immunohistochemistry and confocal microscopy demonstrated that 18 Gy AIR reduces the number of α-SMA+, desmin−ve ISEMF (Fig. 5A) and F480+ve pericryptal macrophages (Fig. 5B). BMASCT restored the α-SMA+, desmin− ISEMF (Fig. 5A) and increased the number of pericryptal and subepithelial macrophages in the lamina propria (AIR+BMASCT, 72±6.4/hpf versus AIR, 15±3.2/hpf; p<0.003; Fig. 5B,C) of irradiated mice. Transplantation of the CD11b−ve fraction of BMASC restored the ISEMF population (Fig. 5A), whereas transplantation of the CD11b+ve fraction exhibited an increase in the number of intestinal macrophages (p<0.006, Fig. 5B,C), which further suggests that transplantation of both CD11b+ and CD11b− fractions restores the ISC niche for RIGS mitigation.

### BMASCT induces secretion of intestinal growth factors and anti-inflammatory cytokines

**Figure 6:**
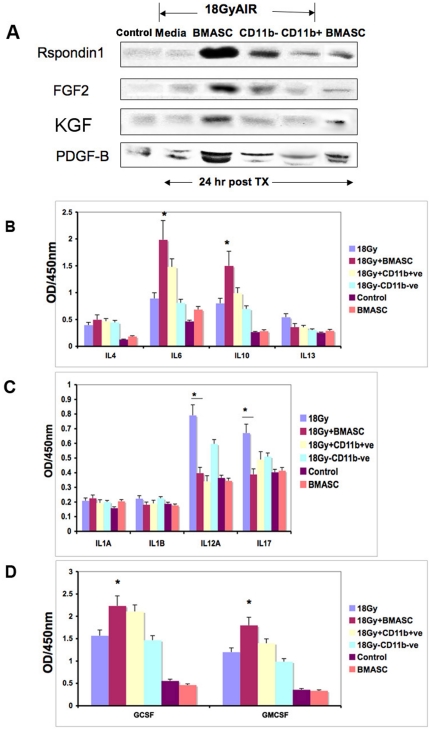
Serum analysis of intestinal growth factors and cytokines. **A.** Immunoblot analysis. An increase in the serum levels of R-spondin1, FGF2, KGF and PDGF-B was noted in AIR+BMASCT cohort compared to AIR. **B–D.** Multi cytokine ELISA. **B.** Anti-inflammatory cytokines, IL6 (p<0.004) and IL10 (p<0.002) levels were significantly increased in the AIR+BMASCT, cohort compared to AIR alone. **C.** Pro-inflammatory cytokines, IL12A (p<0.001) and IL17 (p<0.006) levels were induced in AIR cohort, compared to AIR+BMASCT treated animals (IL12A, p<0.001; IL17, p<0.008). **D.** Myeloid cytokines, GM-CSF (p<0.007) and G-CSF (p<0.006) were increased in AIR+BMASCT group, compared to AIR.

We examined the engraftment and repopulation of the donor cells in various organs by transplanting dipeptidyl peptidase IV (DPPIV)-proficient BMASC in DPPIV-deficient C57Bl/6 host. Although some DPPIV+ve donor cells were noted per intestinal villi upon DPPIV immunohistochemistry (Figure S7 A–B), the majority of the donor cells were lodged in the lungs (Figure S7 C–D). We, therefore, hypothesized that the regeneration and repair of the irradiated intestine is possibly mediated by paracrine growth factors that were secreted by the donor BMASCs. Immunoblot analysis of the serum of animals that received AIR+BMASCT showed an increase in serum levels of R-spondin1, FGF2, PDGF-B and KGF by 2–8 folds at 24 h post-BMASCT, compared to animals that received AIR alone (Fig. 6A). Interestingly, animals that received whole BMT did not show an increase in serum R-spondin1 levels (Figure. S8). While KGF and R-spondin1 can increase the proliferation of intestinal crypt cells [1], [24], FGF2 and PDGF-B could support the growth of endothelial cells [4] and ISEMF [25], respectively in the ISC niche of AIR+BMASC-treated animals.

RIGS is associated with a systemic inflammatory response syndrome (SIRS) resulting from bacterial entry from the denuded gut lumen and resultant endotoxemia [26]. We performed multi-cytokine ELISA in the serum of animals that received AIR alone and compared them with those that received AIR+BMASCT. Compared to untreated controls, there was a significant increase in serum pro-inflammatory cytokines, such as, IL12A (p<0.001), IL17 (p<0.006) in animals that received AIR (Fig. 6C) or AIR+BMT (Figure S9B). BMASCT reduced the secretion of these inflammatory cytokines, while inducing the release of anti-inflammatory cytokines, IL6 (p<0.004) and IL10 (p<0.002) (Fig. 6B) that may dampen the SIRS in RIGS. AIR+BMASCT also increased the levels of serum GCSF (p<0.006) and GMCSF (p<0.007) (Fig. 6D) compared to AIR alone, which could induce macrophage infiltration and activation in the irradiated intestine (Fig. 5B).

Since BMASCT was postulated to modulate the ISC niche, we also examined the expression of mRNA level of intestinal growth factors and inflammatory cytokines from cells isolated from the crypt region. Quantitative RT-PCR analysis of crypt cell mRNA from AIR+BMASCT-treated animals showed several fold increase in expression level of intestinal growth factors, such as, FGF10, KGF, EGF, FGF2, and anti-inflammatory cytokine, IL-10 with BMASCT at 24 hr post-AIR, compared to AIR alone (see Tables S1, S2, S3). While R-spondin1 levels were elevated in the serum, its expression was absent in the crypt region. In contrast to BMASCT, whole BMT had lower expression of intestinal survival and growth factors and chemokines, such as, EGF, FGF10, FGF, IGF1, VEGFa, CSF1, CXCL1 and CXCL12 (Table S1). These results suggested that bone marrow-derived stromal cells could modulate the regenerative signals in intestinal microenvironment.

### Depletion of host macrophages reduces survival of AIR+BMASCT-treated mice

**Figure 7:**
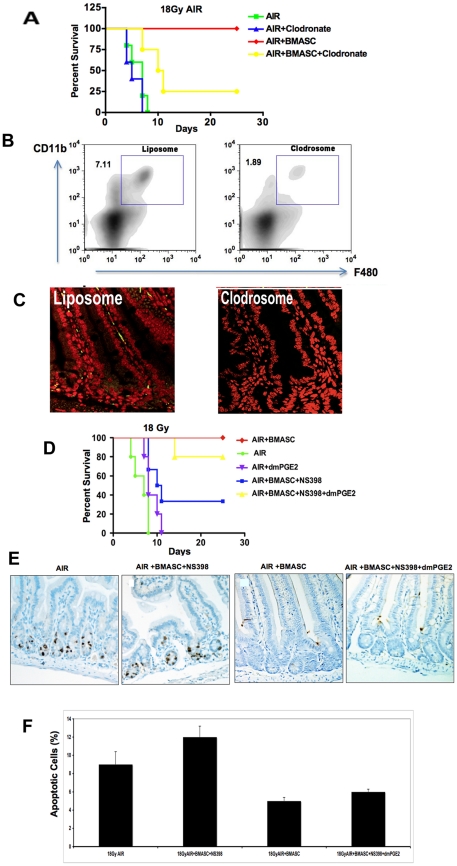
BMASCT promotes signaling cross-talk between macrophages and ISEMF in the ISC niche post-AIR. **A.** Kaplan-Meier survival analysis of animals treated with AIR+BMASCT following depletion of host macrophages by clodrosome. Clodrosome treatment reduced the animal survival after AIR+BMASCT to 25%, indicating host macrophages are needed for mitigation. **B–C.** Flow cytometric (B) and confocal microscopic evaluation (C) of macrophages. Note depletion of host macrophages post-AIR by clodrosome. **D–F.** Inhibition of COX2 reduced the BMASCT mediated mitigation of RIGS. **D.** Kaplan-Meier survival analysis. Administration of COX2 inhibitor, NS398, reduced survival of animals treated with AIR+BMASCT (p<0.008). Survival was improved to 80% with dmPGE2 supplement. **E–F.** TUNEL staining of crypts. AIR+BMASCT inhibited apoptosis in the crypts at day 3.5, which was increased by NS398-mediated COX2 inhibition (p<0.002). Supplementation with dmPGE2 restored the anti-apoptotic effect of BMASCT (p<0.005).

Pericryptal macrophages play an important role in forming synapses with ISC and modulating ISC regeneration [6]. To evaluate the involvement of host macrophages in RIGS mitigation, we depleted them by administering clodronate-filled liposomes (clodrosome) intraperitoneally from day 4 pre-AIR to a week post-AIR. The depletion of macrophages (CD11b+F480+) was verified using FACS analysis of splenocytes and immunohistochemical staining of intestinal sections (Fig. 7B–C). Macrophage depletion reduced the RIGS-mitigating effect of BMASCT with only 25% of the animals surviving after 18 Gy AIR, compared to 100% survival in mice that received AIR+BMASCT (Fig. 7A). This indicated an essential role of host macrophages in the regenerative process of irradiated intestines.

### Prostaglandin E2 (PGE2) is an essential mediator for BMASCT-induced RIGS mitigation

Intestinal macrophages have been implicated in inducing the expression of COX2 for PGE2 synthesis by ISEMF. PGE2 has been known to be involved in selfrenewal and differentiation process of hematopoetic stem cell (HSC). Furthermore, PGE2 increased the homing efficiency of HSCs with the selective induction of short-term-HSC engraftment in murine models [27].

Moreover, it was shown that PGE2 also inhibits the radiation-induced apoptosis of intestinal crypt cells by binding to the EP receptor on ISC [9], [10]. To further elaborate on the cross-talk of pericryptal macrophages and ISEMF in the ISC niche that are replenished after BMASCT and also involvement of PGE2 in repair process, we inhibited PGE2 synthesis with COX2 inhibitor NS398. COX2 inhibition reduced the BMASCT-mediated survival of irradiated animals to 35% (p<0.008), which was restored to 80% with dmPGE2 supplementation (Fig. 7D). Tunnel staining demonstrated that COX2 inhibition significantly increased the percent of apoptotic cell in crypt of animals that received AIR+BMASCT (p<0.002) (Fig. 7 E–F), which was reduced with dmPGE2 supplementation (Fig. 7 E–F).

## Discussion

This is the first demonstration of RIGS mitigation by BMASCT, 24 hours after exposure to high doses of either, single fraction of whole body irradiation (10.4 Gy) or AIR (16–20 Gy). BMASCT restores the ISC niche, including, the pericryptal macrophages, endothelial cells and ISEMF. In contrast to BMT that mitigates radiation-induced hematopoeitic syndrome by donor cell repopulation, BMASCT mitigates RIGS via accelerated regeneration of irradiated host ISC rather than its replacement with donor derived cells. This would require the presence of Lgr5^+^ ISCs, which were noted in crypt for 24 hrs post-AIR, thus affording a time window for effective radio-mitigation. Hence, BMASCT was successful in rescuing animals up to 24 hrs post-radiation but not at later time points.

Since the majority of the donor cells were lodged in the lungs, radiation injury was perhaps mitigated by secreted growth factors. Potential candidates include R-spondin1, KGF, FGF2, PDGF-B, IL-6, IL-10, G-CSF and GM-CSF. Serum R-spondin1 levels increased by 8–10-fold. Human R-spondin1, a 29 kd, 263 amino acid protein that acts as a specific growth factor of intestinal crypt cells [28], has been shown to be a mucosal protective agent in radiation and chemotherapy-induced mucositis [29]. We have demonstrated that R-spondin1 can be radioprotective for RIGS [1]. R-spondin1 binds with high affinity to the Wnt co-receptor, LRP6, and induce phosphorylation, stabilization and nuclear translocation of cytosolic β-catenin, thereby activating TCF/β-catenin-dependent transcriptional responses in intestinal crypt cells [30]. The presence of nuclear β-catenin in the crypt cells of AIR+BMASCT-treated animals could represent R-spondin1-mediated Wnt activation in ISC of these animals. BMASCT also modulated the mRNA expression of several intestinal growth factors in the crypt cells of irradiated intestine. However, R-spondin1 was not expressed in the cells of the crypt region.

BMT can rescue animals that develop primarily a hematopoietic syndrome with exposure to radiation doses ≤8–9 Gy in single fraction. With higher doses of irradiation, intestinal injury sets in and animals cannot be rescued by BMT alone. Although, bone marrow-derived, MSCs contribute to intestinal regeneration and transplantation of these cells ameliorated intestinal injury in murine models of radiation and chemotherapy-induced injury, colitis, and autoimmune enteropathy [16], [18], [31], [32], MSC transplantation alone failed to improve survival of animals exposed to higher irradiation doses (>9.6 Gy) in a single fraction [16], [17], [18]. Our study shows that whole bone marrow transplantation cannot mitigate intestinal injury induced by irradiation (≥10.4 Gy). However, upon amplification of stromal cells in mesenchymal basal medium culture, and transplantation of a combination of CD11b+ macrophages and CD11b− MSC and EPCs could effectively mitigate RIGS. Important differences were noted in the animals that received BMASCT from BMT. In contrast to the AIR+BMT cohort, the AIR+BMASCT cohorts had elevated levels of serum R-spondin1 and expressed various intestinal growth factors in the crypt cells, suggesting a role of stromal cells in secreting growth factors and signals for inducing ISC proliferation in these animals. These stromal cells secrete factors that support the regeneration of the ISC and its niche. Increased serum levels of PDGF-B and FGF2, growth factors for ISEMF and EPC proliferation [25], along with GMCSF and GCSF [33], [34] for macrophage activation support the involvement of BMASC in restoring the ISC niche. Several growth factors that could mediate intestinal regeneration, such as, FGF10, FGF, EGF, IGF1, VEGFa, CSF1 and CXCL12 were induced in the crypt cells in BMASCT-transplanted animals. ISEMF residing throughout the lamina propria and pericryptal region plays a vital role in intestinal structural regeneration [7], [8], [25]. Similarly, submucosal macrophages are activated by the bacterial ligands for Toll-like receptors (TLR) upon bacterial entry through disrupted intestinal mucosa. Thus activated macrophages act as “mobile cellular transceivers” that transmit regenerative signals to ISCs [6]. Crosstalk between host macrophages and ISEMF was necessary for RIGS mitigation by PGE2-mediated inhibition of radiation-induced apoptosis of crypt cells, also noted in other studies [9], [10]. Regenerative role of PGE2 is very well established in hematopoetic system where it was reportedly involved in engraftment as well as survival of transplanted HSCs or cord blood cells [27], [35]. Moreover in embryonic and adult zebrafish model it was shown that PGE2 is required for Wnt-mediated effects on HSC development and can enhance Wnt activity in-vivo [27], [36]. It was quiet evident in our observation that PGE2 has a significant role in BMASCT-mediated amelioration of RIGS. Based upon previous studies [27], [35], it is possible that PGE2 could increase the engraftment of stromal cells. Furthermore, PGE2 from ISC niche may induce Wnt signaling in ISCs, thereby participating in intestinal regeneration [27], [36].

In summary, these experiments point towards a new paradigm for RIGS mitigation, whereby growth factors secreted after BMASCT induce regeneration of the irradiated host crypt progenitors and ISC niche, thereby, accelerating functional recovery of the intestine in RIGS. By reducing the levels of pro-inflammatory cytokines, while inducing anti-inflammatory cytokines, BMASCT also dampens the SIRS in RIGS. Thus, BMASCT provides a platform to discover potential biological agents for mitigation of acute radiation syndromes and for mucosal radioprotection during chemoradiation therapy of abdominal malignancies.

## Materials and Methods

### Animals

Five- to 6-weeks-old male C57Bl/6 (NCI-Fort Dietrich, MD), dipeptidyl peptidase-deficient (DPPIV−ve) (gift from Dr. David Shafritz, Einstein College, Bronx, NY) Lgr5-EGFP-IRES-creERT2 (Jackson Laboratories, Bar Harbor, Maine) mice were maintained *ad libitum* and all studies were performed under the guidelines and protocols of the Institutional Animal Care and Use Committee of the Albert Einstein College of Medicine. The animal use protocol for this study was reviewed and approved by the Institutional Animal Care and Use Committee (IACUC) of Albert Einstein College of Medicine (IACUC approval# 20080703).

### Irradiation

Irradiation was performed on anesthetized mice (intraperitoneal ketamine and xylazine 7∶1 mg/ml for 100 µl/mouse) using a 320 KvP, Phillips MGC-40 Orthovoltage irradiator at a 50 cm SSD with a 2 mm copper filter at a dose rate of 72 cGy/min. We administered WBI (10.4 Gy) or escalating doses of AIR (16–20 Gy) after shielding the thorax, head and neck and extremities and protecting a significant portion of the bone marrow, thus inducing predominantly RIGS.

### BMASC transplantation

Donor bone marrow cells were harvested using sterile techniques from the long bones from C57Bl/6 mice and cultured in MSC basal medium (Cambrex-Lonza, Walkersville, MD) supplemented with 10% heat inactivated FBS, 1% Glutamine, and 1% Penicillin/Streptomycin for 4 days, followed by collection of adherent cells as BMASC. BMASC were then subjected to flow cytometric characterization to determine the percentage of MSC (CD105+CD45−/CD29+CD45−), EPC (CD34+CD133+CD45−) and macrophages (CD11b+ F480+). CD11b+ve and CD11b−ve cells were fractionated using anti-CD11b-magnetic beads (MACS, Miltenyi Biotec, Auburn, CA), following the manufacturer's protocol. Fractionated and whole BMASC (2×10^6^ cells/mice) were injected intravenously via tail vein to C57Bl6 mice at 24 and 72 s hours after irradiation.

### Characterization of RIGS

Animals were sacrificed at 1, 3.5 and 7 days after irradiation for histopathological analysis to examine apoptosis by TUNEL staining, regenerating crypt colonies and villi denudation (Hematoxylin and eosin staining) [1]. To visualize villous cell proliferation, each mouse was injected intraperitoneally with 120 mg/kg BrdU (Sigma-Aldrich, USA) 2–4 hrs prior to sacrifice and mid-jejunum was harvested for paraffin embedding and BrdU immunohistochemistry (Text S1). The crypt proliferation rate was calculated as the percentage of BrdU positive cells over the total number of cells in each crypt. A total of 30 crypts were examined per animal for all histological parameters. A regenerative crypt was confirmed by immunohistochemical detection of BrdU incorporation into five or more epithelial cells within each crypt, scored in a minimum of four cross-sections per mouse. The number of regenerative crypts was counted for each dose of irradiation and represented using the crypt microcolony assay [1], [21], [22].

### Characterization of ISC

Lgr5+ve ISCs were detected in 4% para-formaldehyde-fixed sections from *Lgr5-EGFP-ires-CreERT2* mouse jejunum by examining EGFP expression using confocal microscopy, according to published protcols [3]. GFP expression was also measured by flow cytometry of crypt cells, isolated from *Lgr5-EGFP-ires-CreERT2* mouse intestines, according to method described earlier [23].

### Characterization of ISC niche

ISEMF were stained in formalin-fixed, paraffin-embedded tissue sections for alpha-smooth muscle actin (α-SMA) and desmin using Cy3-conjugated mouse anti-α-SMA (1∶500; Sigma, St. Louis, MO) and rabbit anti-desmin (1∶250; Abcam, Cambridge MA) antibody, respectively, with overnight incubation at 4°C followed by staining with goat anti-rabbit Alexafluor 488 (1∶1000; Molecular Probes, Carlsbad, CA). Pericryptal macrophages were stained by Alexa Fluor488-conjugated, rat anti-mouse, F480 antibody (1∶50; Caltag laboratories, Carlsbad, CA). Images were captured using a Zeiss SP2 confocal microscope at 63× optical zoom and the macrophages were counted by using the VelocitySoft Version 5.0 (Improvision, Waltham, MA) in 10 fields per mice in various cohorts (n = 3).

### Intestinal absorption

Functional regeneration of the irradiated intestines was determined by measuring intestinal absorption by a xylose uptake assay [1], [37]. Briefly, 5% w/v D-xylose solution was administered orally by feeding tube (100 µL/mice, n = 5/cohort), followed by collection of blood 2 hours post-feeding. Plasma xylose levels were measured by a modified micro-method [37].

### Cytokines and growth factors in blood

Intestinal growth factors, R-spondin1, keratinocyte growth factor (KGF), basic fibroblast growth factor (bFGF) and platelet derived growth factor-b (PDGFb) were detected in serum by immunoblotting using goat polyclonal anti-mouse antibodies to R-spondin1 (1∶200; R & D Systems, Minneapolis, MN), KGF (1∶250), bFGF (1∶250) and PDGFb (1∶250). Inflammatory cytokines were measured in the serum using a multianalyte ELISArray kit (SA Biosciences, Fredrick, MD), according to manufacturer's protocol.

### Cytokine and growth factors in crypt cells

To compare the mRNA levels of different growth factors and cytokines in intestine crypt cells from AIR and AIR+BMASCT treated mice, real time PCR were performed using growth factor (cat # PAMM-041) and cytokine (cat # PAMM-011) real time array system from SA Biosciences.

### Macrophage depletion

To deplete macrophages liposomal clodronate (Encapsula NanoSciences, Nashville, TN, USA) (30 mg/kg of body weight) was injected intravenously from day 4 pre-AIR to a week post-AIR. Plain liposome was injected as control. Neither the clodronate filled nor the empty liposomes are considered toxic to the organs.

### Inhibition of COX2

NS-398 (Biomol, Plymouth Meeting, PA) was administered at a dose of 1 mg/kg of body weight (3×/week, ip) started at 1 week prior to AIR. Animals treated with dmPGE2 (Sigma) received a dose of 0.5 mg/kg of body weight (3×/week, ip) started at 1 week prior to AIR.

### Kaplan-Meier Survival analysis

Mice survival/mortality in different treatment group was analyzed by kaplan-Meier as a function of radiation dose using Graphpad Prism-4.0 software for Mac.

### Statistical analysis of digital images

Sampling regions were chosen at random for digital acquisition for data quantitation. Digital image data was evaluated in a blinded fashion as to any treatment. A two-sided student's t-test was used to determine significant differences between experimental cohorts (P<0.05) with representative standard errors of the mean (SEM).

## Supporting Information

Figure S1 **Flowcytometric characterization of freshly isolated bonemarrow cells for expression of MSC specific (A) (CD105+ CD45−) (B) (CD29+ CD45−), (C) macrophage specific (CD11b+F480+) and (D) EPC specific (CD133+ CD34+CD45−) markers.** It was noted that bone marrow cell were primarily enriched with CD45+ hematopoetic cells (A–B).
(TIF)

Figure S2 **Blood count was performed with the help of ANTECH DIAGNOSTICS (LAKE SUCCESS, NY) to evaluate the effect of abdominal irradiation (AIR) on hematopoesis.** Absence of any significant changes in (A) differential count and (B) number of RBC and among the irradiated and transplanted group in comparison to untreated control group suggested AIR could not affect the bone marrow.
(TIF)

Figure S3 **Expression of different MSC surface markers CD105, CD29, CD44, SCA1 in BMASC population.** Staining for IgG isotype fluorescence was used as a control. Isotype control for CD105, CD29, CD44 and SCA-1 are rat IgG2a, hamster IgG, rat IgG2bΚ and rat IgG2aΚ respectively.
(TIF)

Figure S4 **Flowcytometric charaterization of CD11b−ve (A–B) and CD11b+ve (C–D) BMASC population for CD105 and CD29 (MSC marker) expression.** It was noted that CD11b−ve BMASC population was primarily enriched with CD105 and CD29 positive cells.
(TIF)

Figure S5 **BMASC transplantation significantly increases crypt depth compared to AIR control.**
(PDF)

Figure S6 **Kaplan-Meier survival analysis.** Mice (n = 15) receiving first dose of BMASC at 72 h post AIR follwed by second dose failed to mitigate RIGS in contrast to BMASCT at 24 h follwed by 72 hr second where 100% survival were noted.
(TIF)

Figure S7 **Transplanted BMASC were primarily detected in intestine and lung.** BMASC from DPPIV positive wild type mice were transplanted to DPPIV negative mice exposed to AIR. (**A&C**) DPPIV immunohistochemistry followed by confocal micrscopic analysis. DPPIV positive BMASC (stained green) were found primarily in the lung (**C**) and intestine (**A**). Nucleus was stained with DAPI and pseudo colored with red. (**B&D**) Quantification of engrafted DPPIV+ve cells. Significantly higher number of engrafted cells in lung (p<0.002) (**B**) and in intestine (p<0.004) (**D**) was noted at 1day post AIR compared to 3.5 day post AIR. Confocal microscopic images (63×) were magnified 2.3× and presented in inset. The number of DPPIV positive cells were counted using volocity soft version 5 (Improvision). Based on the intensities, number of cells were determined by scoring at least 10 fields from each animal (n = 3). Resolution of the images were same for both experimental and control groups.
(TIF)

Figure S8 **Immunoblot analysis of intestinal growth factors in serum.** An increase in the serum levels of R-spondin1, FGF2, KGF and PDGF-B was noted in AIR+BMAST treated animals, compared to animals that received AIR+BM or AIR alone.
(TIF)

Figure S9 **A–B. Multi cytokine ELISA.** **A.** Anti-inflammatory cytokines, IL6 (p<0.004) and IL10 (p<0.002) levels were significantly increased in the AIR+BMASCT-treated animals, compared to AIR alone. Induction of anti-inflammatory cytokine IL6 (p<0.007) and IL10 (p<0.005) was also observed in the animals treated with AIR+CD11b+ve BMASCT. Transplantation of freshly isolated bone-marrow cells could not increase the anti-inflammatory cytokine level. **B.** AIR+BMASCT reduces the pro-inflammatory cytokine levels (IL12A, IL17), compared to AIR alone. Transplantation of freshly isolated bone-marrow cells could not reduce the pro-inflammatory cytokine level compare to AIR alone. **C.** AIR+BMASCT induces the GMCSF and GCSF levels compared to AIR alone. Transplantation of freshly isolated bone-marrow cells did not induce the GMCSF and GCSF level.
(TIF)

Figure S10 **BMSCT maintains villus length after radiation injury.** Low magnification images (10×) of jejunal cross-sections showed the reduction of villi length and thickness (H&E staining) with the decrease in Brdu positive crypt cells in irradiated cohort (18 Gy AIR) compared to 18 Gy+BMASC group.
(TIF)

Table S1 **qPCR analysis of different growth factor mRNA level in intestinal crypt cells.** RT+BMASCT treated group showed significant increase in mRNA level of growth factors compared to RT cohort.
(DOC)

Table S2 **qPCR analysis of inflammatory cytokine in intestinal crypt cells.** RT+BMASCT treated group showed significant increase in mRNA level of anti-inflammatory cytokine level compared to RT cohort.
(DOC)

Table S3 **Median survival time of animals exposed to 18 Gy AIR and 10.4 Gy WBI followed by cell transplantation.** Please note the clear difference of median survival time of the animals exposed to 18 Gy AIR compared to 10.4 Gy WBI.
(DOC)

Text S1 (DOC)
