# Supplementary material for: Bone Marrow Stromal Cell Transplantation Mitigates Radiation-Induced Gastrointestinal Syndrome in Mice
Source: PLoS One. 2011 Sep 15;6(9):e24072. doi: 10.1371/journal.pone.0024072 (PMC3174150; doi:10.1371/journal.pone.0024072)
Supplement: Table S1 — qPCR analysis of different growth factor mRNA level in intestinal crypt cells. RT+BMASCT treated group showed significant increase in mRNA level of growth factors compared to RT cohort. (DOC) [file pone.0024072.s011.doc]

| **GROWTH FACTORS** | **RT +BMASCT VS RT** log2(Fold Change) | **RT+BM VS RT**  log2(Fold Change) |
| --- | --- | --- |
| EGF | 13.70 | -2.11 |
| FGF10 | 124.59 | -2.01 |
| FGF2 | 11.02 | 4.71 |
| KGF | 2.19 | 5.22 |
| IGF1 | 106.21 | -2.97 |
| HGF | 3.27 | 1.09 |
| VEGFa | 2.16 | -3. |
| CSF1 | 4.83 | -5.00 |
| CSF3 | 4.25 | 1.13 |
| CXCL1 | 28.34 | 2.04 |
| CXCL12 | 67.80 | -1.34 |
