# Supplementary material for: Bone Marrow Stromal Cell Transplantation Mitigates Radiation-Induced Gastrointestinal Syndrome in Mice
Source: PLoS One. 2011 Sep 15;6(9):e24072. doi: 10.1371/journal.pone.0024072 (PMC3174150; doi:10.1371/journal.pone.0024072)
Supplement: Table S2 — qPCR analysis of inflammatory cytokine in intestinal crypt cells. RT+BMASCT treated group showed significant increase in mRNA level of anti-inflammatory cytokine level compared to RT cohort. (DOC) [file pone.0024072.s012.doc]

| **CYTOKINE AND CYTOKINE RECEPTOR** | **BMASCT+RT VS RT**  log2(Fold Change) | **RT+BM VS RT**  log2(Fold Change) |
| --- | --- | --- |
| IL10 | 2.10 | 2.16 |
| IL10Ra | 2.32 | 2.02 |
| IL11 | 2.23 | -3.24 |
