# Supplementary material for: Bone Marrow Stromal Cell Transplantation Mitigates Radiation-Induced Gastrointestinal Syndrome in Mice
Source: PLoS One. 2011 Sep 15;6(9):e24072. doi: 10.1371/journal.pone.0024072 (PMC3174150; doi:10.1371/journal.pone.0024072)
Supplement: Table S3 — Median survival time of animals exposed to 18 Gy AIR and 10.4 Gy WBI followed by cell transplantation. Please note the clear difference of median survival time of the animals exposed to 18 Gy AIR compared to 10.4 Gy WBI. (DOC) [file pone.0024072.s013.doc]

| Treatment | **Median survival time (Days)** | |
| --- | --- | --- |
|  | 18Gy AIR | 10.4 Gy WBI |
| IR | 6+1.2 | 10+1.4 |
| IR + GROWTH MEDIA | 6+1.8 | 7+1.1 |
| IR+BMNAC | 12+1.6 | 7+1.8 |
| IR+BM | 11+1.2 | 10+1.3 |
