# Supplementary material for: Bone Marrow Stromal Cell Transplantation Mitigates Radiation-Induced Gastrointestinal Syndrome in Mice
Source: PLoS One. 2011 Sep 15;6(9):e24072. doi: 10.1371/journal.pone.0024072 (PMC3174150; doi:10.1371/journal.pone.0024072)
Supplement: Text S1 — (DOC) [file pone.0024072.s014.doc]

**Methods:**

**BMASC Characterization**

For characterization of mesenchymal stem cell, single cell suspensions of BMASCs were stained with CD105 conjugated with PE (BD Biosciences, San Diego, CA) or CD29 (conjugated with PE, BD Biosciences) and CD45 conjugated with FITC (BD Biosciences). To determine the EPC population, BMASCs were stained with CD34 (pacific blue) (ebioscience, San Diego, CA) CD45 (FITC) CD133 (APC) (ebioscience). For determination of monocyte/macrophage population BMASC were stained with CD11b (PE) (BD Biosciences) and F480 (FITC) (BD Biosciences). BMASC were acquired with the LSRII flow cytometer (BD Biosciences). The acquired data was analyzed with FlowJo v.7.1 (Treestar Inc, Ashlaand, OR, USA) software.

**Macrophage depletion from BMASC**

CD11b+ve, macrophages were depleted from BMASC using anti-CD11b-magnetic beads (MACS, Miltenyi Biotec, Auburn, CA), following the manufacturer’s protocol. Briefly, BMASC were incubated with CD11b micobeads for 15 mins at 4-8^o^C. Then cell suspension was washed by adding 10x labeling volume of buffer per 10^8^ cells and centrifuged at 300xg for 10 mins. Supernatant was pipette off and 500 μl buffer was added per 10^8^ cells. Then MS magnetic column (Miltenyl Biotec) was placed in MACs separator (magnet) and prepared by rinsing the column with 500 μl of rinsing buffer. Cell suspension was applied onto the column. Unlabelled cells [CD11b-ve] were passed and collected in the flow through. Column was washed three times with 500 μl of buffer. Column was then removed from the magnetic field and placed in a suitable collection tube. 1 ml of buffer was added in the column and immediately flushed with plunger supplied with the column to separate the magnetically labeled cells from the column. CD11b positive and negative population was confirmed by flowcytometry using anti CD11b antibody (Miltenyl Biotec).

**Histology**

The intestine of each animal was dissected, washed in PBS to remove intestinal contents and the jejunum was fixed in 10% neutral buffered formalin prior to paraffin embedding. Paraffin embedded tissue was processed and cut into 5μm sections for hematoxylin and eosin and immunohistochemical staining. All hemotoxylin and eosin (Fisher Scientific, Pittsburgh, PA) staining was performed at the Histology and Comparative Pathology Facility in the Albert Einstein Cancer Center**.**

**BrdU immunohistochemistry & measurement of Crypt Proliferation rate**

Mouse was injected intraperitoneally with 120mg/kg BrdU (Sigma-Aldrich, USA) 2-4 hrs prior to sacrifice and mid-jejunum was harvested for paraffin embedding and BrdU immunohistochemistry. Tissue sections were routinely deparaffinized and rehydrated through graded alcohols and incubated overnight at room temperature with a biotinylated monoclonal BrdU antibody (Zymed, South Francisco, CA). Nuclear staining was visualized using Streptavidin-peroxidase and diaminobenzidine (DAB) and samples were lightly counterstained with hematoxylin. Jejunum from mice, not treated with BrdU, was used as a negative control. Murine crypts were identified histologically as reported earlier[[3]](#_ENREF_1). High (400-600X) magnification digital photographs of crypts were taken at (Zeiss AxioHOME microscope) and crypt epithelial cells (paneth and non-paneth) intestinal sections were examined using ImageJ software. Cells with brown-stained nuclei from DAB staining were classified as BrdU positive. The crypt proliferation rate was calculated as the percentage of BrdU positive cells over the total number of cells in each crypt.

**Immunohistochemistry**

For immunohistochemical staining of formalin-fixed, paraffin-embedded tissue sections, endogenous peroxidase activity was blocked for 30 min with methanol containing 0.3% H_2_O_2_. Antigen retrieval was performed by heating slides in pH 6.0 citrate buffer at 100°C for 20 min in a microwave oven at 500 watts. Non-specific antibody binding was blocked for 20 minutes by incubation with serum free protein block (Dako, San Antonio, CA). Sections were further incubated with target specific primary and fluorescent conjugated secondary antibodies. Images were captured using a Zeiss (Thornwood, NY) SP2 confocal microscope equipped with cooled CCD cameras at 63 optical zoom. Resolution of the images was same for both experimental and control groups.

**Crypt isolation**

In brief, isolated mouse small intestines were chopped into around 5 mm pieces and were washed with cold PBS. The tissue fragments were incubated in 2 mM EDTA with PBS for 30 min on ice followed by centrifugation. After removal of EDTA medium, the tissue fragments were vigorously suspended by using a 10-ml pipette with cold PBS and fractionated thereafter by centrifugation to get the crypt-enriched sediment. The crypt-enriched fraction was further suspended with PBS with vigorous dispersions and subjected to centrifugation. The supernatant enriched with crypts were collected and passed through a 70-mm cell strainer (BD Bioscience) to remove residual villous material. Isolated crypts were centrifuged at 150–200g for 3 min to separate crypts from single cells. Isolated crypts were incubated in culture medium for 45 min at 37^o^C, followed by trituration with a glass pipette. Dissociated cells were passed through cell strainer and were acquired with the LSRII flow cytometer (BD Biosciences). The acquired data was analyzed with FlowJo v.7.1 (Treestar Inc, Ashlaand, OR, USA) software.

**Xylose Absorption assay**

xylose uptake assay among different treatment group (n = 5/group) was performed, at various time points (1, 3.5, 7 and 10 days) after irradiation. A 5% w/v solution of D-xylose (100μl/mouse) in deionized water was given orally by feeding tube and animals were sacrificed at 2hrs post administration of D-xylose. Blood samples were collected in heparinized blood collection tubes (BD Biosciences, San Jose, CA). 50 μL of serum sample was added to 5ml of phloroglucinol (1,3,5-trihydroxybenzene, Sigma Chemical Co., St. Louis, MO) color reagent (0.5 g of phloroglucinol, 100 ml glacial acetic acid and 10 mL of conc. HCL) heated to 100^o^C in a water bath for 4 min to allow optimum color development. After equilibration to room temperature, sample absorption was determined with the aid of a spectrophotometer set at a wavelength of 554 nm.

**Detection of transplanted BMASC**

DPPIV deficient mice received AIR. The BMASC were transplanted as mentioned earlier. Frozen sections were prepared by freezing tissue in OCT (Tissue Tek) To analyze the engraftment of DPPIV positive transplanted cells in situ 5 uM cryostat sections were incubated with streptavidin conjugated anti mouse DPPIV (R&D systems, McKinley Place NE, Minneapolis) for overnight at 4^0^C and followed by incubation with streptavidin alexafluor 488. The nucleus was stained with DAPI. Images were captured using a Zeiss SP2 confocal microscope equipped with cooled CCD cameras at 63 optical zoom. The DPPIV positive cells were counted by using volocity soft version 5 (Improvision). Based on the intensities, number of cells was determined by scoring at least 10 fields from each animal (n=3). Resolution of the images was same for both experimental and control groups.

**Immunoblotting of serum R-spondin1, KGF, FGFb, PDGFb**

Blood was drawn from the retro-orbital plexus and serum was isolated by centrifugation at 10,000 rpm for 5 min. Serum protein concentration was determined by Bradford assay kit (Bio-Rad Laboratories, Hercules, CA). Approximately 100μg of protein was subjected to 14% SDS-PAGE, followed by electroblotting onto polyvinylidene difluoride membranes. The blot was blocked with 5% skim milk in Tris-buffered saline (10mM Tris-HCl (pH 7.4), 150 mM NaCl, 0.05% Tween 20) followed by incubation with primary antibody (1:200 dilution), goat polyclonal anti mouse R-spondin1 (R & D Systems, Minneapolis, MN), KGF (1:250), FGFb (1:250), PDGFb (1:250) and then with secondary antibody (1:500 dilution), horseradish peroxidase (HRP) conjugated bovine anti-goat antibody (Santa-Cruz Biotechnology, Inc., Santa Cruz, CA). The blots were developed using Enhanced Chemiluminence assay (Amersham Pharmacia Biotech, Inc, Piscataway, NJ).

**ELISA array of inflammatory cytokines**

Blood was drawn from the retro-orbital plexus and serum was isolated by centrifugation at 10,000 rpm for 5 min. Serum protein concentration was determined by Bradford assay kit (Bio-Rad Laboratories, Hercules, CA). Approximately 100μg of serum protein was subjected for ELISA using multianalyte ELISArray kit (SA Biosciences, Fredrick, MD) according to manufacturer’s protocol.

**Isolation of cells along the crypt-villus axis**

Cells along the crypt-villus axis were isolated following the protocol described by Chang et al [1] and Ferraris et al [2]. Briefly the entire small intestine (duodenum to terminal ileum) was removed and flushed once with phosphate buffered saline (PBS). The small intestine was then tied off at one end, and filled to distension with PBS prior to closing the open end followed by incubation at 37°C for 15 minutes in 15 ml of buffer B (96 mM NaCl, 1.5 mM KCl, 27 mM Na-citrate, 8 mM KH2PO4, and 5.6 mM Na2HPO4, pH 7.3), and for 10 minutes in 15 ml of buffer C (PBS plus 1.5 mM EDTA, 0.5 mM dithiothreitol, and 1 mg/ml bovine serum albumin), in a shaking 37°C incubator. Detached enterocytes were collected (Fraction 1) at the end of 15 minutes incubation and 15 ml of fresh buffer C added to the tissue. This procedure was repeated four more times, the steps lasting 25, 25, 25 and 30 minutes, respectively (Fractions 2, 3, 4 and 5), for a total of 120 minutes of incubation time. Viability and morphology of isolated cells were identified by trypan blue using light microscopy. Fractions 3-5 were further subjected to RNA isolation as they are reportedly known to contain crypt region.

**RNA Isolation and cDNA synthesis.**

Isolated crypt cells were lysed using RLT buffer from RNeasy Mini Kit (Qiagen, Valencia, CA) and 1% beta-mercaptoethanol mix. Qiagen's protocol for the RNeasy Mini Kit with on-column DNA digestion was used to isolate RNA from the lysates. Isolated RNA samples were subjected to cDNA synthesis using first strand synthesis kit from SA Biosciences (Frederick, MD) and according to manufacturer protocol and stored at −80°C prior to use until further use for real time PCR.

**References**:

1. Chang J, Chance MR, Nicholas C, Ahmed N, Guilmeau S, Flandez M, Wang D, (2008) Byun DS, Nasser S, Albanese JM, Corner GA, Heerdt BG, Wilson AJ, Augenlicht LH, Mariadason JM. Proteomic changes during intestinal cell maturation in vivo. J Proteomics;71:530-46.

2. Ferraris RP, Villenas SA, Diamond J. (1992) Regulation of brush-border enzyme activities and enterocyte migration rates in mouse small intestine. Am J Physiol;262:G1047-59.

3. Bhanja P, Saha S, Kabarriti R, Liu L, Roy-Chowdhury N, et al. (2009) Protective role of R-spondin1, an intestinal stem cell growth factor, against radiation-induced gastrointestinal syndrome in mice. PLoS One 4: e8014.
